# iHOPerator: user-scripting a personalized bioinformatics Web, starting with the iHOP website

**DOI:** 10.1186/1471-2105-7-534

**Published:** 2006-12-15

**Authors:** Benjamin M Good, Edward A Kawas, Byron Yu-Lin Kuo, Mark D Wilkinson

**Affiliations:** 1 The James Hogg iCAPTURE Centre for Cardiovascular and Pulmonary Research, Providence Health Care/University of British Columbia, St. Paul's Hospital, Rm. 166, 1081 Burrard St. Vancouver, British Columbia, V6Z 1Y6, Canada

## Abstract

**Background:**

User-scripts are programs stored in Web browsers that can manipulate the content of websites prior to display in the browser. They provide a novel mechanism by which users can conveniently gain increased control over the content and the display of the information presented to them on the Web. As the Web is the primary medium by which scientists retrieve biological information, any improvements in the mechanisms that govern the utility or accessibility of this information may have profound effects. GreaseMonkey is a Mozilla Firefox extension that facilitates the development and deployment of user-scripts for the Firefox web-browser. We utilize this to enhance the content and the presentation of the iHOP (information Hyperlinked Over Proteins) website.

**Results:**

The iHOPerator is a GreaseMonkey user-script that augments the gene-centred pages on iHOP by providing a compact, configurable visualization of the defining information for each gene and by enabling additional data, such as biochemical pathway diagrams, to be collected automatically from third party resources and displayed in the same browsing context.

**Conclusion:**

This open-source script provides an extension to the iHOP website, demonstrating how user-scripts can personalize and enhance the Web browsing experience in a relevant biological setting. The novel, user-driven controls over the content and the display of Web resources made possible by user-scripts, such as the iHOPerator, herald the beginning of a transition from a resource-centric to a user-centric Web experience. We believe that this transition is a necessary step in the development of Web technology that will eventually result in profound improvements in the way life scientists interact with information.

## Background

User-scripts are programs, typically written in JavaScript, that are installed inside web-browsers. They manipulate the content of specified sets of Web pages prior to their display in the browser. The name 'user-script' may be slightly misleading as a typical user of a Web browser will not likely write user-scripts (but see [1] for work on making this more feasible). The name might more appropriately be 'user-side-scripts' to convey the notion that the script operates within the user's browser and that its installation and activation is under the user's control. For brevity and to stay in alignment with common terminology, we will use 'user-scripts' throughout the rest of the text.

User-scripts can be used to perform tasks including, but not limited to: automatically adjusting style sheets, stripping unwanted advertisements, integrating the content of multiple Web resources, or introducing novel visualizations. Anyone capable of writing JavaScript can write and share user-scripts that alter the content displayed on any Web page. By writing or locating a suitable user-script, for example in a public repository such as userscripts.org [2], and installing it in their browser, users gain unprecedented control over the content that is ultimately displayed in their browser window. User-scripts thus offer an immediate mechanism by which the Web browsing experience can be shifted from its current resource-centred pattern of control towards a more user-centred view.

Here we introduce the iHOPerator – a user-script designed to provide an enhanced, customized view of the iHOP website, a key bioinformatics resource describing proteins, their properties, and the relationships that hold between them. We describe how the iHOPerator script generates and embeds a novel visualization of the contents of the iHOP Web pages and extends the content of those pages with information gathered from related, external Web resources. We conclude with a discussion of the potential implications of user-scripts, describing their relationship with the emerging Semantic Web in the life sciences.

### iHOP

**Figure 1:**
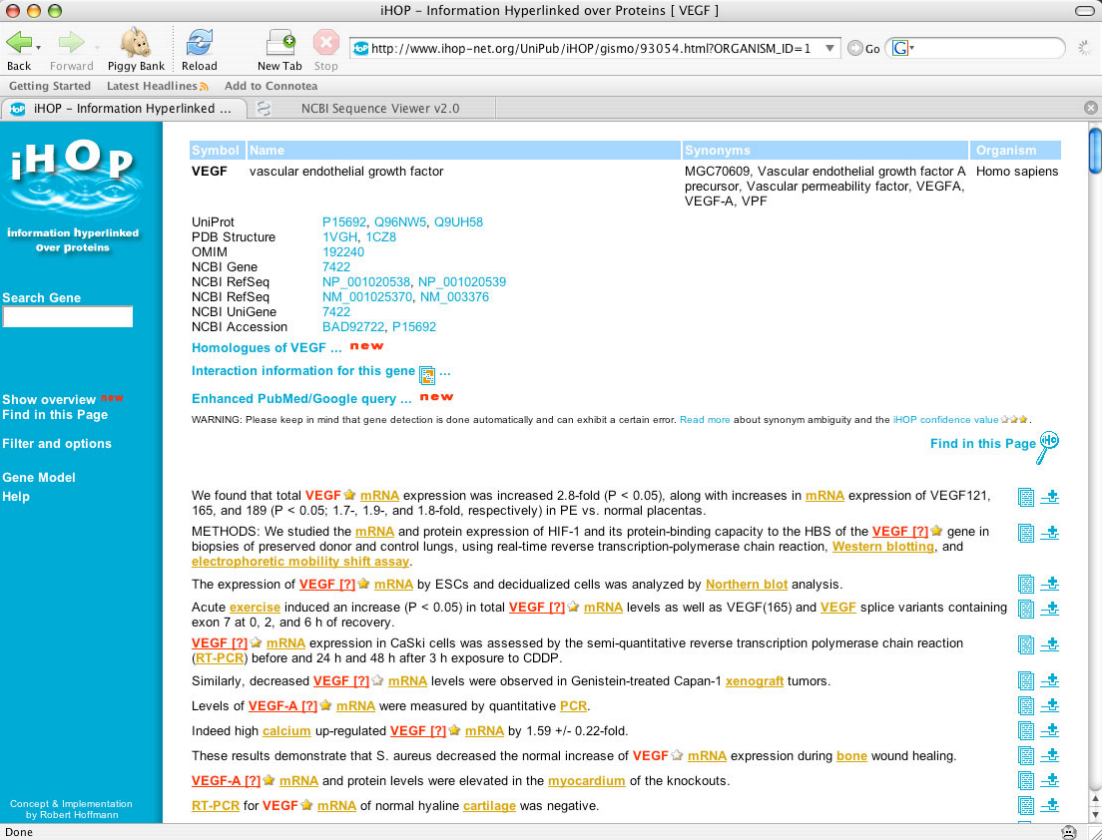
**Default iHOP page displaying the defining information for VEGF**. The default iHOP gene-focused Web page without the enhancements provided by the iHOPerator script. The page is displaying the defining information for the gene VEGF. The top of the page displays alternate names while the bottom (extending well past the area that can be displayed in the figure) provides extractions from the text of abstracts associated with the gene.

The iHOP database provides information about proteins that have been automatically associated with PubMed abstracts [3-5]. Using the iHOP website [6], it is possible to browse through the literature using hyperlinks that associate abstracts to one another using co-occurring genes. After identifying a gene of interest, a user may navigate to a page that contains the "defining information" for the gene. This information consists of the gene's names in different databases, its source organism, and a potentially very long list of snippets of text that have been extracted from abstracts associated with the gene (Figure 1).

### Tag clouds

Tag clouds are visually-weighted renditions of collections of words ('tags') that are used to describe something [7]. Tags in a cloud are sized, organized and coloured so as to illustrate aspects of the relationship between each tag and the entity that it describes. Tag clouds have recently gained popularity in 'social-tagging' applications such as Flikr [8], Connotea [9], and del.icio.us [10] because they provide a mechanism through which untrained users can quickly visualize the dominant features of voluminous databases and because they provide a visually based navigation paradigm that is complementary to text search and operates naturally over non-hierarchically organized information systems.

## Implementation

The iHOPerator is a user-script, a JavaScript that can be embedded in a Web browser such that it processes the contents of visited Web pages prior to their presentation to the user. Though a user-script may be instructed to process any set of Web pages, (*e.g*. those from a particular domain) the iHOPerator is focused specifically on the gene-information pages of the iHOP website.

### GreaseMonkey

At this time, most user-scripts require extensions to Web browsers such as GreaseMonkey [17] for Mozilla's Firefox, Creammonkey [18] for Apple's Safari, and Turnabout [19] for Microsoft's Internet Explorer. Though user-scripts for each of these browsers are written in JavaScript, there are no accepted standards for user-script extensions and thus scripts written for one browser may or may not work in another browser. As user-scripts become more popular, standardization efforts are likely to emerge that will improve script/browser interoperability; for the moment however, the iHOPerator is built for Firefox and is thus dependent on the GreaseMonkey extension for its operation.

The GreaseMonkey/Firefox combination was chosen for this project because both are cross-platform, actively developed, open source, and because GreaseMonkey was the first and is still the most widely used browser extension for housing user-scripts. We utilize GreaseMonkey to add a tag cloud to pages describing genes on the iHOP website by processing the HTML and JavaScript present on those pages prior to presentation in the browser. As well, we extend the content of the website by utilizing the GreaseMonkey API to retrieve content from external HTTP-accessible resources.

## Results

The purpose of the iHOPerator user-script is to enhance the user's experience when visiting the iHOP Web page. It does this by generating a tag cloud visualization of some of the information presented on the gene-information Web pages and by integrating additional content acquired from PubMed[11] and the Kyoto Encyclopedia of Genes and Genomes (KEGG)[12].

### iHOPerator tag clouds

**Figure 2:**
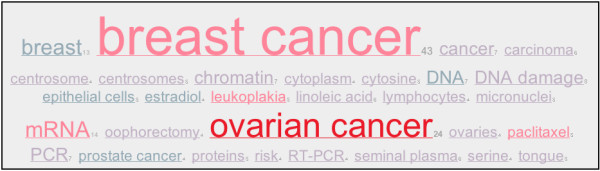
**A tag cloud built from MESH terms associated with Brca1**. This tag cloud was built automatically using the iHOPerator user-script. It is composed of MESH terms extracted from abstracts associated with the gene Brca1 (in mouse). Colour (redness) correlates with the impact factor of the journals where the term occurs. Size correlates with the number of times the term occurs in association with the gene – in this case Brca1.

**Figure 3:**
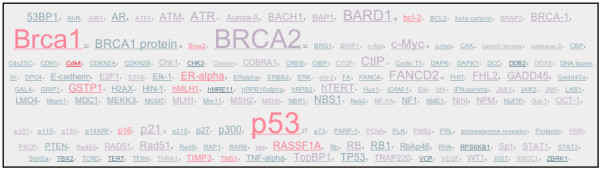
**A tag cloud built from genes related to Brca1**. This tag cloud was built automatically using the iHOPerator user-script. It is composed of gene names extracted from abstracts associated with the gene Brca1 (in mouse). Colour (redness) correlates with the impact factor of the journals where the gene name occurs. Size correlates with the number of times the related gene name occurs in association with the gene in question – in this case Brca1.

**Figure 4:**
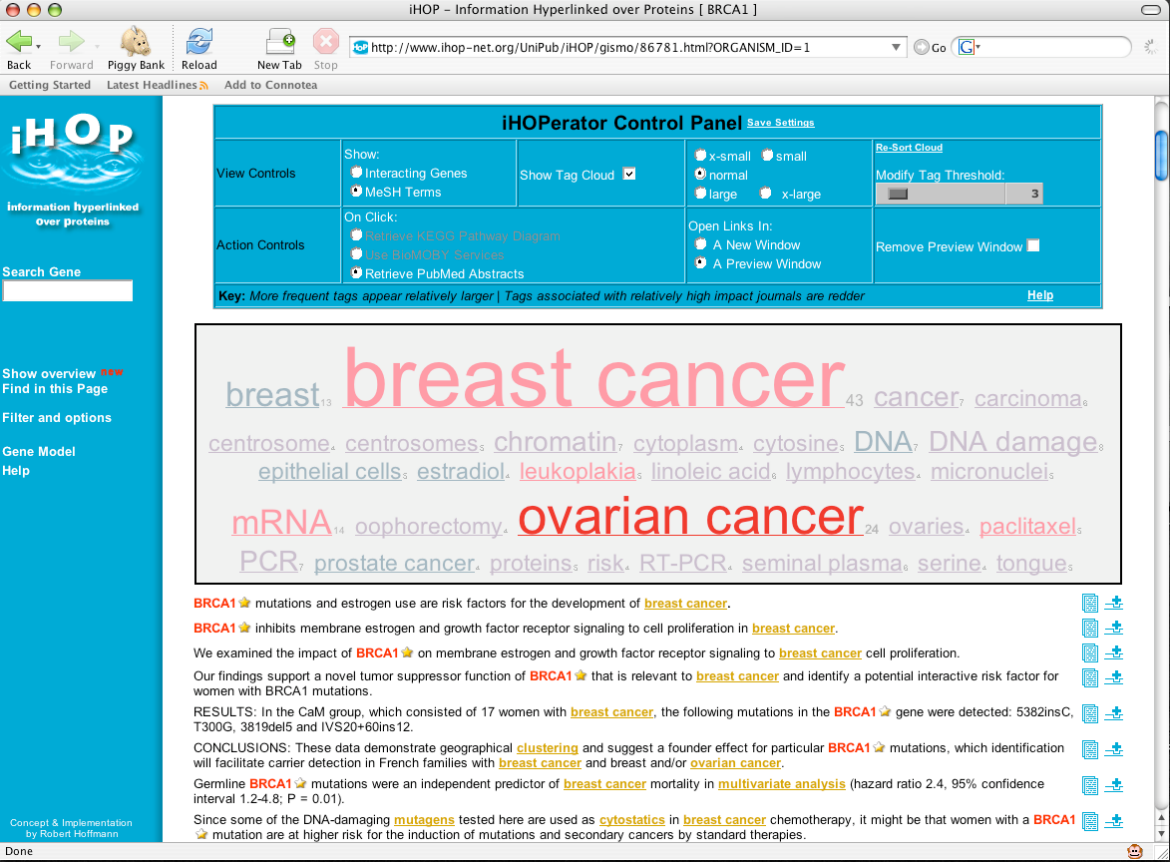
**The iHOP webpage enhanced by the iHOPerator user-script**. The iHOP webpage after it has been enhanced with the iHOPerator user-script. Compare with Figure 1. The Web page now includes a tag cloud composed of MESH terms from abstracts associated with the gene Brca1 in mouse as well as a panel of controls for manipulating the new visualization. The number of terms used to build the cloud, the scale of the fonts used, the presence or absence of the cloud on the page, and the actions taken when the user clicks on an element of the cloud are all under the user's control.

The iHOPerator script produces tag clouds based either on MESH keywords from the abstracts associated with a gene or from other genes that iHOP identifies as interacting with a gene. For example, (Figure 2) shows a tag cloud generated using MESH terms gathered from abstracts associated with the gene Brca1 and (Figure 3) shows a tag cloud composed of genes related to Brca1. In both clouds, the size of each tag is used to display the frequency of occurrence of that tag (gene or keyword) in the context of abstracts associated with Brca1 and colour is used to highlight the impact factor of the journals in which the tags appear. From the user's perspective, these tag clouds appear to be embedded directly within the iHOP Web page (Figure 4).

The process of generating the tag clouds works as follows:

1. Extract tags (MESH keywords or interacting genes) embedded in the HTML of the page. (This is greatly facilitated by the presence of XML mark-up of these entities provided by the iHOP website).

2. Count the number of occurrences of each tag

3. Calculate a score for the tag based on its relative frequency in the page.

4. Collect the impact factor assigned to each abstract and associate it with the appropriate tag. (Once again, this is facilitated by XML mark-up in the iHOP page).

5. Find the average impact factor associated with each tag.

6. Produce the HTML for the cloud by

a. Assigning each tag to a predefined Cascading Style Sheet class that is associated with a particular size and colour that is determined by the frequency of occurrence of the tag in the page and the average impact factor of the journals associated with the tag occurrences respectively.

b. Sorting the tags alphabetically.

The iHOPerator script also allows the user to customize the interface by selecting different ranges for the font sizes in the cloud and by specifying whether iHOPerator-generated content should be hidden, display in another window, or display within the iHOP Web page.

### iHOPerator integration of third-party content

**Figure 5:**
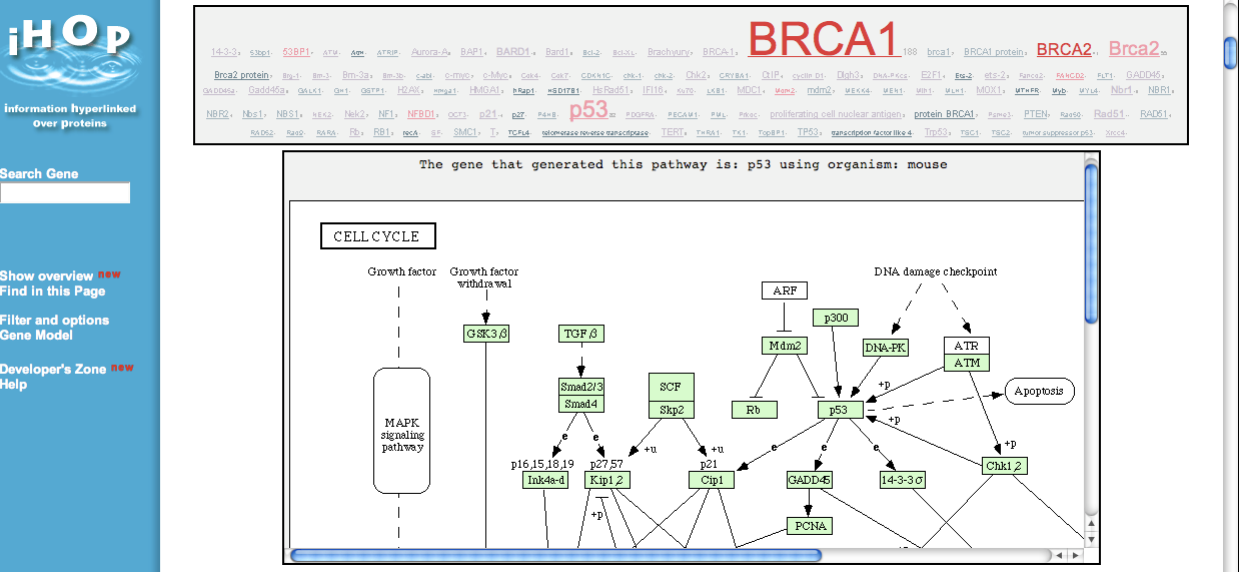
**The iHOP webpage for IRF-3, enhanced with a tag cloud and a pathway diagram using the iHOPerator user-script**. The iHOPerator user-script is shown providing access to a KEGG pathway diagram containing the gene IRF-3 within the context of the iHOP website. The diagram was retrieved as a result of a mouse-click on 'IRF-3' in the tag cloud.

Aside from the tag-cloud based visualization (produced entirely using JavaScript operating within the browser), a key feature of the iHOPerator script is its ability to acquire and display third-party content related to the gene in the same browser-context. For example, the script utilizes GreaseMonkey's built in support for AJAX (Asynchronous JavaScript and XML) to execute an asynchronous HTTP request that invokes a BioMoby [13] Web service workflow stored as a Java servlet that, when possible, provides KEGG pathway diagrams containing the gene of interest (Figure 5). The script also makes it possible for the user to access relevant external websites using an embedded IFRAME element. This allows the user to view the abstracts associated with the gene and/or MESH term of interest or to initialize a Web service browsing session using the Gbrowse Moby [14] BioMoby client application that originates with a gene selected from the cloud. Without the iHOPerator, each of these activities would require that the user find the additional resources themselves, learn how to use them, cut and paste search terms into them, and of course, navigate away from the iHOP website.

## Related work

Within the bioinformatics domain, only a few examples of user-scripts appear to exist so far. At the time of this writing, only two were listed at the primary global repository [2] and one was identified via Web search [15]. Both scripts listed on [2] facilitate the addition of bookmarks to articles listed in PubMed [11] to similar science-focused social bookmarking systems, Connotea [9] and CiteULike [16]. In the other, Pierre Lindenbaum provides a script that generates a TreeMap [17] visualization of Connotea reference collections [15].

## Discussion

At present, Web browsers are the dominant technology used to satisfy the information gathering and visualization needs of life scientists. In their current form, browsers provide users with the ability to retrieve information from widely distributed sources, but essentially no means to integrate information from multiple sources and only a very constrained set of operations for manipulating the display of that information. Given the distributed nature of information on the Web and the diversity of user requirements in interacting with that information, this situation is unsatisfactory.

In most current implementations, Web browsers facilitate information transfer between only two parties – the resource provider, who determines all information presented, all links to external resources, and nearly all manner of visualizing that information; and the consumer, who essentially can only control which page they choose to view next. The typical Web browsing experience can thus be characterized as *resource-centric *because everything that the user sees on a Web page is governed entirely by the resource provider.

By introducing an additional layer of processing that occurs only at the discretion of the user (by choosing whether or not to install a given script), user-scripts offer a way to effect a transition towards a *user-centric *browsing experience. Though it has always been possible for the technically skilled to engineer their own software for processing Web content (*e.g*. the notorious 'screen-scraping' characteristic of early bioinformatics [18]), the arrival of popular browser extensions such as GreaseMonkey marks the beginning of a fundamental change in the way end-users can interact with the Web. Empowered with the ability to easily embed scripts directly into their browser and to find such scripts in public repositories, Web users can now more actively make decisions about what Web content they see and how that content is presented.

Despite its intriguing, paradigm-shifting nature, the user-script concept is not without its problems. Because Web content is still primarily provided as HTML, user-scripts must process HTML in order to function. This is problematic for two reasons: 1) HTML is not designed for knowledge or data representation and hence is difficult to parse consistently and 2) HTML representations may change frequently even when the underlying data does not. The former makes it challenging to write effective user-scripts, particularly scripts that are intended to operate over multiple Web pages. The latter makes these scripts brittle in the face of superficial changes to their inputs and thus potentially unreliable [18]. Since information on the Web is currently provided primarily as HTML, alterations to the structure of this content are frequent and necessary results of the need to keep the browsable interfaces up to date. To alleviate these problems, it would clearly be beneficial if the underlying data could be exposed in a manner that was independent of its HTML representation

The potential value of separating content from presentation provides motivation for the Semantic Web [19] initiative and the standards for the annotation of Web resources, such as the Resource Description Framework (RDF)[20] and the Web Ontology Language (OWL)[21], that have recently emerged from it. With these standards in place, content providers are encouraged to provide a representation of their data for visualization (HTML) in parallel with an additional representation of their data for machine-interpretation (RDF/OWL). This would enable those who wish to utilize the content in novel ways to process the more stable, machine-readable representations while remaining unaffected by visual modifications to the associated websites. Though widespread adoption of Semantic Web standards by the community may, in principle, enable the creation of powerful, user-centred applications that go beyond the capabilities of user-script enabled browsers [22], this process is occuring very slowly [23] and the problems faced by life scientists in gathering, integrating and interpreting information on the Web are pressing. In their current form, user-scripts, such as the iHOPerator, provide an immediate means to address these needs and thus should be more widely exploited to this end.

## Conclusion

By adding the iHOPerator user-script to their browser, users gain access to 1) a novel method of visualizing and navigating the defining information about genes on the iHOP website and 2) enhancements to that information that are gathered automatically using external resources such as PubMed and KEGG. The iHOPerator thus provides an extension to the iHOP website that demonstrates how user-scripts can be used to personalize and to enhance the Web browsing experience in a biological context.

User-scripts represent a small, but immediate and useful step in the direction of a user-centred rather than a resource-centred Web browsing experience. In contrast to other proposed routes to achieving this goal, they offer a mechanism that can be effected immediately using existing resources and representations to provide end-users with a straightforward way to exert greater control over what and how they see on the Web.

## Availability and requirements

• Project name: iHOPerator

• To install: Go to the project homepage and follow the installation instructions

• Project homepage: 

• Operating system: any OS that supports the Mozilla Firefox Web browser

• Programming languages: JavaScript

• Other requirements: JavaScript enabled Firefox Web browser, GreaseMonkey Firefox extension, Internet connection

• License: FreeBSD

## Authors' contributions

BMG instigated the project and drafted the manuscript. EAK wrote all of the software. BYK developed the project website and provided intellectual input throughout the project. MDW provided substantial advice and guidance during all phases of the project and assisted in the drafting of the manuscript. All author's read and approved the final manuscript.
